# Diversity, Equity, and Inclusion Statements and Initiatives on US Residency Program Websites: A Cross-Sectional Study

**DOI:** 10.1227/neuprac.0000000000000129

**Published:** 2025-03-11

**Authors:** Maria Bederson, Naomi Bennett, Daniel Herrick, Odette Harris

**Affiliations:** * Department of Neurosurgery, Stanford University, Palo Alto, California, USA; ‡ Current Affiliation: Carle Illinois College of Medicine, University of Illinois Urbana-Champaign, Urbana-Champaign, Illinois, USA; § Current Affiliation: Rush Medical College, Chicago, Illinois, USA

**Keywords:** DEI, Diversity, equity, and inclusion, Disability, Neurosurgery residency

## Abstract

**BACKGROUND AND OBJECTIVES:**

Diversity, equity, and inclusion (DEI) are increasingly relevant in academic neurosurgery and workforce recruitment. This study assessed DEI statements and initiatives available on US neurosurgery residency program (NRP) websites, recognizing this as one objective measure of DEI efforts.

**METHODS:**

NRP websites were identified and selected based on defined inclusion and exclusion criteria. Websites were examined for DEI statements, dedicated DEI language, information about current residents and faculty, disability assistance, and subinternships and residency interviews financial support.

**RESULTS:**

Among 110 eligible websites, 35 NRPs (32%) mentioned DEI on their home page or mission statement. Of these, 24 programs (22%) had a specific diversity statement. Gender, race, and ethnicity were mentioned in 13 (54%), 11 (46%), and 8 statements (33%), respectively. Disability, sexual orientation, LGBTQ+, and minority statuses were noted in 5 (21%), 8 (33%), 2 (8%), and 2 statements (8%). Among the 86 programs without a diversity statement, 11 (13%) mentioned DEI, 2 (2%) discussed gender, 2 (2%) mentioned disability and gender, and 1 (1%) covered race, ethnicity, LGBTQ+, and minority statuses. Eight programs (7.2%) offered scholarships for visiting fourth-year students, but none provided financial support for residency interviews.

**CONCLUSIONS:**

Dedicated DEI statements and initiatives available on NRP websites represent a tangible metric of DEI representation in neurosurgery. Our study showed substantial variation in the depth and specificity of DEI representation on NRP websites and provides a benchmark on this topic. Strategies aimed at enhancing DEI representation might enhance the future success of diversification of the neurosurgery workforce.

**ABBREVIATIONS:**
DEI: diversity, equity, and inclusion
NRP: neurosurgery residency program
NTP: neurosurgery training programs

Diversity is defined as “the practice or quality of including or involving people from a range of different social and ethnic backgrounds and of different genders, religions, etc.”^1^ Diversity, equity, and inclusion (DEI) are an extension of this term and are often used in academia to represent an institutional commitment to equal opportunity for individuals from different backgrounds and with different needs.^2^

The need for a diverse physician workforce reflecting its population has been highlighted. Increased diversity in health care correlates with minimized racial health disparities, improved accuracy of clinical decision-making, and greater financial returns.^3^ In addition, diversity in the surgical workforce has been associated with increased patient-reported satisfaction, improved clinical outcomes, increased adherence to medical recommendations, and increased participation in clinical research studies.^4-7^ Patient representation in the healthcare workforce may even lead to decreased postoperative mortality, as highlighted in a recent study on gender concordance between patient and surgeon.^8^

Although there have been efforts to increase diversity in health care, surgical specialties, including neurosurgery, have made modest progress.^9,10^ The neurosurgical workforce is predominantly male and White, with women representing 6% to 10% and people of color representing 4%.^10,11^ Neurosurgical residency programs (NRP) mirror these statistics, with women representing 17% to 19% of the workforce, one of the lowest representations among medical specialties,^11,12^ Latinx residents comprising 5.8% and Black residents 4.8%.^13,14^ To create a more inclusive environment, efforts aimed at expanding diversity for recruitment, retention, and success are sought.

Residency program websites provide accessible preliminary information to medical students while they explore the neurosurgery application process.^15^ Therefore, residency website DEI statements can provide valuable insight about a program's commitment to diversity and its definition.^9^ Recognizing that websites are readily available to the public, the aim of this study was to analyze the current presence of DEI statements and initiatives posted on US NRP websites as a measurable metric to provide a cross-sectional benchmark.

## METHODS

### Neurosurgery Residency Program Identification

The researchers performed a cross-sectional observational study of the current NRP websites, listed by the American Association of Neurological Surgeons.^16^ Inclusion criteria included the following: (1) neurosurgery training programs (NTP) based in the United States, (2) NTP currently accepting residents, and (3) NTP with a functioning website. Exclusion criteria included the following: (1) NTP outside the United States; (2) NTP offering fellowship but not residency; (3) NTP no longer functioning, “sun-downing”; and (4) NTP without a functioning hyperlink.

### Data Collection and Analysis

NRP websites were searched by two independent researchers between October 1st and November 13th, 2023. The primary outcome measure was the presence of a department-specific or residency-specific diversity statement. For each website, binary response variables were used to denote the presence or absence of a diversity statement. If a statement was identified, it was further reviewed for the presence of the key components of diversity as outlined by its definition in the introduction. Specifically, the statements were searched for the inclusion of the following terms: “gender,” “race,” “ethnicity,” “religion,” “LGBTQ+,” “disability/ability,” and “minority.” These terms were selected to contextualize the described research within the existing literature investigating similar topics.^17,18^ Diversity statements were further analyzed with an open-source word cloud generator to gain key insights regarding the language used in these statements, including frequency.^19^ For NRP websites on which no statement was found, websites were searched for keywords including “diversity,” “equity,” “inclusion,” “race,” “ethnicity,” “women,” “gender,” “LGBTQ+,” “minority,” and “disability.” Binary response variables were used to denote the presence or absence of each of these terms.

Secondary outcome measures for all websites included the presence of a disability coordinator mentioned on any page of the residency websites and information regarding current residents and faculty, including total number, presence of photographs, extensive biographies beyond education history, and provision of diversity statistics. Data were collected through binary response variables or total number in the case of number of residents or number of faculty. Websites were further searched for mention of Sub-Internship scholarships and for residency interview funding or scholarships. When available, the information regarding scholarships and funding was stored and summarized. All data were collected on Google Sheets and stored on Excel (Version 16.68, Microsoft Corporation).

The characteristics of programs with department-specific or residency-specific DEI statements were analyzed for trends. Number of faculty and residents at programs with department-specific residency statements were compared with programs without statements. Program funding was also investigated through publicly available NIH grant funding amounts within their respective neurosurgical departments.^20^

### Statistical Analysis

Statistical significance for differences in average program faculty and residency size was conducted through one-way analysis of variance. Continuous variables are reported as mean ± SD or percentages of the total N. Statistical analyses were performed with Python (Python 3.12.6). A two-sided *P* value of <.05 was considered statistically significant.

### IRB

Approval from the Institutional Review Board was not necessary because the data are publicly accessible, do not involve human subjects, and do not include any sensitive information. Patient consent was not obtained because the study did not involve patient level data.

## RESULTS

**FIGURE 1.**
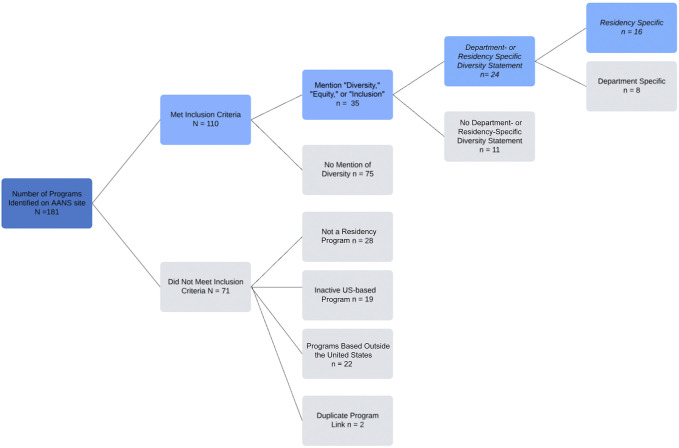
Neurosurgery training programs search strategy and diversity, equity, and inclusion web posting.

Of the 181 listed NTP, 110 met inclusion criteria for this study, as summarized in Figure 1.

**FIGURE 2.**
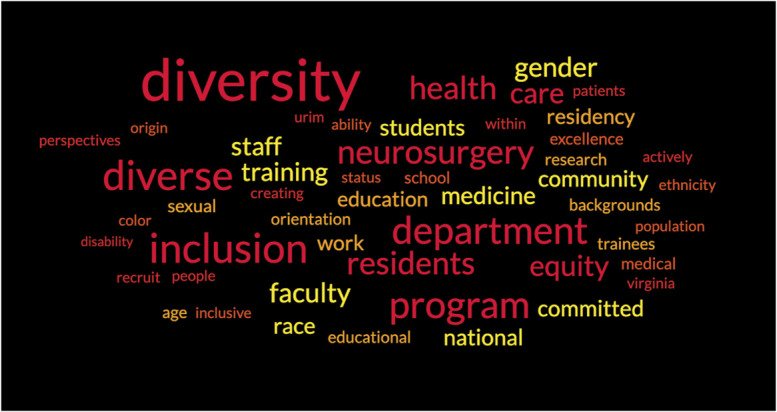
Residency-specific 50 most common words.

The terms “diversity,” “equity,” and/or “inclusion” were mentioned on 35 of 110 (32%) NRP websites. The 50 words with highest frequency are shown in Figure 2. Unique (n = 16) or department-specific (n = 8) DEI statements were found in 24 NRP websites (23%) (Figure 1). Diversity statements highlighted the terms “gender,” “race,” “sexual orientation,” “ethnicity,” “disability,” “women,” “LGBTQ+,” and “minority” in order of decreasing frequency (Table). Among the 24 NRP with a dedicated diversity statement, gender, race, and ethnicity were mentioned in 13 (54%), 11 (46%), and 8 statements (33%), respectively. Disability or physical ability, sexual orientation, LGBTQ+, and minority were mentioned in 5 (21%), 8 (33%), and 2 statements (8%). Of the 86 NRP lacking a diversity statement, 11 (13%) mentioned “diversity,” “equity,” or “inclusion” on their residency homepage or mission statement. Two programs (2%) mentioned “disability” or “physical ability,” “gender,” and “women.” One program (1%) mentioned “race,” “ethnicity,” “LGBTQ+,” and “minority” (Table).

**TABLE.** Key Components of DEI Language Posted on Neurosurgery Residency Websites

DEI keyword	Neurosurgery residency program–specific statement (n = 16)	Neurosurgery department–specific statement (n = 8)	All residency/department-specific DEI statements (n = 24)	Websites without a residency/department-specific diversity statement (n = 86)	All programs (N = 110)
DEI	16 (100%)	8 (100%)	24 (100%)	11 (13%)	35 (32%)
Gender	11 (69%)	2 (25%)	13 (54%)	2 (2%)	15 (13%)
Race	9 (56%)	2 (25%)	11 (46%)	1 (1%)	12 (11%)
Sexual orientation	8 (50%)	0	8 (33%)	0	8 (7%)
Ethnicity	5 (31%)	3 (38%)	8 (33%)	1 (1%)	9 (8%)
Disability/physical ability	4 (25%)	1 (13%)	5 (21%)	2 (2%)	7 (6%)
Women	2 (13%)	0	2 (8%)	2 (2%)	4 (3%)
LGBTQ+	2 (13%)	0	2 (8%)	1 (1%)	3 (3%)
Minority	1 (6%)	1 (13%)	2 (8%)	1 (1%)	3 (3%)

DEI, diversity, equity, and inclusion.

Disability coordinators were mentioned on 7 websites (6.2%). Most programs included photographs of their current residents (97%), and almost half included extended resident biographies (48%). Most departments included photographs of their faculty (97%), and more than half included extended faculty biographies (61%). Three programs (2.6%) provided diversity statistics. Eight programs (6.2%) mentioned scholarships for visiting fourth year medical students completing a subinternship, 7 of which were neurosurgery department specific. Based on publicly available data, no program offered scholarship or financing for residency interviews.

**FIGURE 3.**
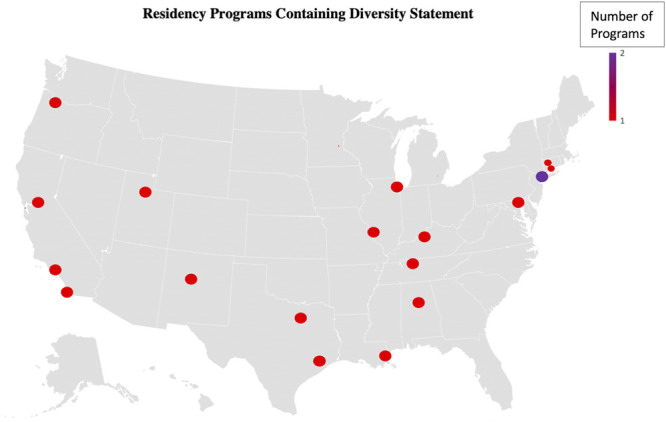
Geospatial representation of US Residency Programs with a diversity statement posted on their websites.

NRPs providing diversity statements had a significant larger department regarding number of residents (16.8 vs 13.5 residents; *P* = .0235, CI 95 (0.0074, 0.0721) but not number of teaching faculty (23.9 vs 20.5 faculty; *P* = .2656, CI 95 (0.1920, 0.3550). Programs with residency-specific diversity statements were clustered in California (21%) and, otherwise, showed no geospatial trend and were distributed across the country in cities of varying sizes (Figure 3). Most programs with residency-specific diversity statements received NIH funding within their neurosurgical department (N = 20, 72%).^20^

## DISCUSSION

### Key Results

To the best of our knowledge, this is the first study evaluating dedicated DEI statements and initiatives available on US NRP websites. Residency program websites are vital sources of information to help future neurosurgeons make informed decisions about which training environments might best fit them. Our cross-sectional study highlights an opportunity to measure and improve diversity within the neurosurgery workforce, as only one-third of NRP websites (32%) mentioned diversity, equity, or inclusion.

Although DEI commitment can manifest in many forms, our study used this specific approach to review training program websites informed by previous research.^17,18^ Given the novelty of this research, we hope that it will serve as a foundation for further investigation about DEI practices within neurosurgery.

### DEI Statements

Diversity statements provide insight into a program's commitment to DEI throughout the recruitment and training process of residency. They are a dedicated space for programs to communicate their support for specific minority groups and promote dedicated resources.^21^ The content of the statement is relevant because it provides specificity to an otherwise broad definition of diversity. The statements searched in this study focused on key components of race, ethnicity, gender, minority status, LGBTQ+, and disability. Programs with residency-specific diversity statements often promoted student interest groups related to gender, sexual orientation, ethnicity, and culture. This is important because when diversity statements are paired with tangible resources and diversity-related initiatives, there has been a measurable impact on the recruitment, retention, and success of a diverse workforce.^22^

Diversity statements are only one method programs can use to demonstrate their commitment to DEI. Other means of promoting DEI are vital.^23^ Photographs and extended biographies of faculty and current residents along with department diversity statistics are also powerful means for communicating a program's commitment to DEI.^24^ Future applicants can use this information to find common ground with members of the department, supporting their ability to feel a sense of belonging to a departmental community and make meaningful connections. Although almost all the programs searched in this study included resident and faculty photographs on their websites, only half included extended resident biographies, detailing what each resident contributes to the department outside of their academic pedigree. Only three programs included diversity statistics, which represents an area for improvement.

### Disability Assistance

Although race and gender are commonly supported areas of diversity, disability status among neurosurgery faculty and residents is scarcely discussed. Although at least 12% of the US population reports having a disability, the prevalence of US practicing physicians reported disability is as low as 3%.^25,26^ Most physicians with disabilities report working in medical schools, nonteaching hospitals and as *locum tenens*, and only 0.5% report working in a surgical specialty.^26^ Among nonmedical companies, hiring persons with disabilities benefits company performance.^27^ Paralleling reports showing benefit from minorities being treated by minority physicians,^28^ concordance in disability status between physicians and patients with disabilities could also improve disparities in care.^29^ This is especially true in neurosurgery where many patients have visible or invisible disabilities secondary to the pathology being treated. Our study shows that 6% of NRP websites have disability key words on their web side, suggesting an area of improvement while updating NRP websites.

### Financial Support

Residency programs can also demonstrate a commitment to DEI by deploying resources to support socioeconomically disadvantaged applicants and trainees. The costs associated with required neurosurgical subinternships and interviews are demanding. In addition, most subinternship applications have an associated fee. This application fee combined with the cost of living for the duration of the 4-week subinternship program and overnight stay for an interview may pose financial barriers for applicants and weaken diversity. Existing literature reports a mean cost of $10 300 ± $5170 for the entire application process including subinternships, applications, and second looks.^30^ This cost is comparable with that associated with application into other competitive surgical subspecialties and significantly higher than nonsurgical specialties.^31^ Other surgical subspecialties, such as plastic, vascular, and thoracic surgery, have a higher representation of financial support for underrepresented minorities in medicine completing subinternship, with one study reporting as high as 62.1% of thoracic surgery programs providing monetary support for this cause.^32,33^

Our study found that only 8 of 110 neurosurgery residency programs (7%) offered scholarships for visiting fourth year medical students. No program publicly offered scholarship for residency interviews. The authors are aware through personal communication of only one residency program, which initiated a pilot for interview financing in the 2024 Match. This was an anonymized, inclusive, no-bar for entry opportunity for all individuals who were invited for an interview. Preliminary data show that approximately 40% of applicants used this financing service, indicating its significance.

Financial factors, including mounting debt, are a known factor in the decision-making process of medical students' career choices, and a lack of resources dedicated to supporting students with economic disadvantage may contribute to professional homogeneity.^34^ Providing financial resources to neurosurgery applicants can help with recruiting a diverse class of future neurosurgeons.^34^

### Legal Implications

It is important to acknowledge the legal context within which DEI statements are created and published. Since the US Supreme Court banned the use of race-conscious admissions programs in higher education, also known as Affirmative Action, there has been an increasing wave of state-specific legislation prohibiting DEI efforts. Currently, the authors are aware of 10 states in which anti-DEI–related bills have been signed into law, and at least 15 states where similar bills have been introduced.^35^ This shift significantly affects the ability of residency programs within these states to include DEI statements on their websites or to engage in DEI initiatives in a public-facing manner. This reality underscores the tension between institutional commitment to DEI and national, and state-level legislative actions, which may inhibit efforts to foster diversity in medical education and practice.

### Limitations

This was a cross-sectional observational study with inherent limitations including lack of causation, selection bias, and human factors. To address these limitations, attention was paid to avoid discussion which attempts to establish causation for the presence or absence of DEI efforts on a residency website page. To avoid selection bias, all sites were searched by independent researchers at all stages of data collection and reviewed by a blinded researcher. Independent researchers practiced searching non-neurosurgical sites together as a template to ensure accurate redundancy. Despite this methodology, the possibility of human error cannot be removed entirely. The researchers also acknowledge that the NRP website is only one location by which NPR communicate with future applicants. Although the authors are aware of one DEI effort, discussed above, which is not available on a public-facing website, it is possible that additional efforts publicized through direct communication with potential applicants, social media, or in-person events are not captured by this study. These incentives may therefore be unknown to prospective applicants. As discussed above, the legal circumstances in each state may also play a role in the availability of public-facing support for DEI incentives.

## CONCLUSION

Including diversity information on residency program websites is one way that programs can demonstrate their commitment to recruiting and training a diverse group of future neurosurgeons and may be a key step in improving the current gap in representation increasingly reported in the neurosurgical workforce. Despite the current political climate and state-level restrictions on DEI initiatives, continuing to prioritize and publicize diversity efforts remains essential for fostering an inclusive environment in the field that reflects the population. The results of our cross-sectional study provide the first benchmark of the DEI statements and initiatives posted on NRP websites. These data suggest several possible strategies to follow while furthering the depth of existing DEI efforts and focus. First, providing granular data including statistics of the department workforce composition might be desirable especially for those programs where diversity already exists. Our study showed that only 3 of 110 programs are currently providing this information. Second, working at creating DEI statements and/or initiative to post on the program's website seems an opportunity for most NRP to remain competitive in recruitment and retention of their workforce. Third, disability assistance and financial support might further enhance other existing DEI efforts and more realistically attract diversity. Further studies are necessary to assess whether the strategies outlined above will effectively advance DEI in neurosurgery. Hopefully, these can spark from collaborative efforts among neurosurgical residency training programs and/or organized neurosurgery with longitudinal data for objective analysis.

## COMMENTS

The authors describe the paucity of DEI statements on the websites of neurosurgery programs in the United States. They nicely show that DEI statements are more likely to be published by programs in distinct locations. DEI statements are becoming commonplace for many businesses, so at times can be overlooked. However, statements of inclusivity can be meaningful in fields with a lack of diversity such as neurosurgery. Attempts to convey an overall departmental culture of openness or willingness to change helps to recruit from a group of the “best applicants” and not just certain applicants. The politicization of diversity statements, fear of being viewed as “woke,” and state laws prevent certain institutions from adoption of DEI statements. These fears or limitations can be overcome by conveying inclusivity and creating an environment of psychological safety that organically recruits a diverse workforce. Having a diverse group of residents who feel included generates a certain energy that reverberates throughout the institution. These are the small but meaningful steps that it will take to transform our field.

**Maryam Rahman**

*Gainesville, Florida, USA*
